# Weak and Dynamic GNSS Signal Tracking Strategies for Flight Missions in the Space Service Volume

**DOI:** 10.3390/s16091412

**Published:** 2016-09-02

**Authors:** Shuai Jing, Xingqun Zhan, Baoyu Liu, Maolin Chen

**Affiliations:** School of Aeronautics and Astronautics, Shanghai Jiao Tong University, No. 800 Dongchuan Road, Shanghai 200240, China; lightning95@sjtu.edu.cn (S.J.); abao-liu@163.com (B.L.); cml@sjtu.edu.cn (M.C.)

**Keywords:** GNSS, adaptive Kalman filter, INS-assisted navigation, maximum likelihood estimation, space service volume, Doppler frequency estimation

## Abstract

Weak-signal and high-dynamics are of two primary concerns of space navigation using GNSS (Global Navigation Satellite System) in the space service volume (SSV). The paper firstly defines a reference assumption third-order phase-locked loop (PLL) as the baseline of an onboard GNSS receiver, and proves the incompetence of this conventional architecture. Then an adaptive four-state Kalman filter (KF)-based algorithm is introduced to realize the optimization of loop noise bandwidth, which can adaptively regulate its filter gain according to the received signal power and line-of-sight (LOS) dynamics. To overcome the matter of losing lock in weak-signal and high-dynamic environments, an open loop tracking strategy aided by an inertial navigation system (INS) is recommended, and the traditional maximum likelihood estimation (MLE) method is modified in a non-coherent way by reconstructing the likelihood cost function. Furthermore, a typical mission with combined orbital maneuvering and non-maneuvering arcs is taken as a destination object to test the two proposed strategies. Finally, the experiment based on computer simulation identifies the effectiveness of an adaptive four-state KF-based strategy under non-maneuvering conditions and the virtue of INS-assisted methods under maneuvering conditions.

## 1. Introduction

The demand for reducing the burden of ground TT&C (Tracking, Telemetry and Command) stations and surveying vessels stimulates the development of precise orbit and attitude determination using GNSS. On the other hand, to take full advantage of the service capacity of GNSS, more flight missions from different space agencies equipped with onboard GNSS receiving terminals will utilize satellite navigation when operating in the space service volume (SSV) [1]. Nevertheless, the volume covered from a height of 3000 km to 36,000 km is difficult for GNSS applications. It should be noted that the altitude of 3000 km is defined as the boundary of the terrestrial service volume (TSV) and SSV, but the altitude (>20,000 km) above the GPS constellation is the most challenging environment for satellite navigation. A research satellite of the Max-Planck Institut für Extraterrestrische Physik, Equator-S, equipped with a dual antenna Motorola Viceroy receiver was launched in December 1997 into a geostationary transfer orbit (GTO) by the German Space Agency (DLR). Its in-orbit experiment proved the reception of GPS signals above the GPS orbit, at an altitude of 34,000 km, is possible [2], but the quality of the physical signals and the data contents were not good enough for spacecraft in-orbit navigation.

The main problems of navigation above the GPS constellation concentrate on two aspects: (1) insufficient signal availability and poor dilution of precision (DOP); and (2) weak signal processing with high dynamic stress and poor ranging accuracy. Both of them degrade the robustness and reliability of GNSS space service. The first problem is mainly related to signal visibility, while the second one is related to signal power. For the state of the art, the former can be improved by an interoperable SSV based on the development of multi-GNSSs interoperability [3], and we specified the GNSS SSV characterization and service performance in terms of four GNSS constellations in our previous research publication [4], but for the second problem, the technical matters are definitely more complicated, which will be the discussion topics in this work.

In normal conditions, the sensitivity of a GNSS receiver and its dynamic performance interact with each other. Fortunately, there are few terrestrial GNSS terminal manufactures that claim both high receiver sensitivity and desirable dynamic performance simultaneously. However, for SSV users, the situation is quite different; herein, the issue about how to process weak GNSS signals with high dynamic stress have to be dealt with. Comparatively, the carrier tracking loop is more likely to lose lock than the code tracking loop in weak-signal and high-dynamic environments. Thus, a third-order phase-locked loop (PLL) is taken as the receiver reference assumption model (RRAM) for space applications. According to automatic control principle [5], the third-order loop is only sensitive to jerk rather than velocity and acceleration. Although the jerk for non-maneuvering space vehicle is quite small, the jerk for orbital maneuvering mission can be as high as 4 g/s (g = 9.8 m/s^2^ is the Earth’s acceleration of gravity) or more. It means that the performance of the SSV RRAM should be analyzed quantitatively.

In order to keep the third-order loop stable, the coherent integration time (CIT) and loop noise bandwidth (LNBW) are designed with upper limitation. By calculating the loop measurement errors caused by thermal noise and dynamic stress respectively, the maximal bearable jerk under different carrier-to-noise density ratio (C/N_0_) can be determined. Obviously, the loop of SSV RRAM is likely to lose its lock in harsh environments, for instance, when the C/N_0_ is lower than 30 dB-Hz and the jerk is over 4 m/s^3^ at the same time.

Owing to this reason, an adaptive four-state Kalman filter (KF)-based algorithm is presented with the intention of maximizing the signal processing performance of closed loop (CL) form. On the basis of estimating the state of code phase and the third-order state vector of the carrier phase, the principle about how to regulate the filter gain matrix to vary the equivalent LNBW is introduced in this work. Furthermore, the relationship between noise and adaptive bandwidth is also discussed.

Actually, the CL form uses a feedback path to make corrections of code phase and carrier frequency errors. In contrast, open loop (OL) tracking updates the local code phase and carrier frequency estimates flexibly, not entirely dependent upon the feedback path [6]. Aided by supplementary measurements, the OL scheme is free of loop instability. Thus, it is suggested that an OL tracking design could be an alternative to be adopted in SSV user receivers to overcome the dilemma of settling the issues of weak-signal and high-dynamics together.

Various OL tracking methods have been developed up to now. A US patent (Patent No: US 6633255B2) [7] proposed four kinds of carrier frequency measuring approaches, based on a frequency doubler, a frequency discriminator, a block phase estimator, and a channelized filter, respectively. Some scholars demonstrated quasi-open loop architecture to update the frequency of the local oscillator (LO) every several epochs, instead of updating frequency estimations at each epoch [8], which can be recognized as an interim scheme between closed loop and open loop. Other literatures released an idea of FFT-based frequency-domain tracking [9], which is a novel high-sensitivity tracking method developed to perform accurate frequency parameter estimation by processing the spectral peak line and adjacent lines [10]. Without doubt, an INS-assisted open loop tracking strategy is another effective method to find the true signals with high dynamics and high noise level [11]. However, all of these methods are derived on the basis of some assumption that disregarding the coupling effect between the carrier phase and Doppler frequency. Then the technical matter comes down to put forward an improved methodology to generate the carrier phase and Doppler frequency estimates decoupled with each other for the LO.

This paper incorporates a GNSS orbit propagator with INS-assisted open loop tracking together to obtain the aiding information about the Doppler frequency. With the purpose of updating the Doppler frequency estimation that is insensitive to the corresponding carrier phase estimation, a non-coherent maximum likelihood estimator is used to eliminate the coupling relationship in this paper. A new gradient function from the two-dimensional log-likelihood cost function for code delay and Doppler frequency is first established, and the new cost function is totally independent of the carrier phase tracking error. Then we can get the gradient and Hessian expressions of this new cost function with respect to the Doppler frequency. When the frequency correction is computed with the gradient divided by the Hessian, the equation does not contain the component of carrier phase and proves the cancellation of the coupling effect. This paper illustrates the implementation block diagram of the open loop tracking structure using the maximum likelihood estimation (MLE) method, and introduces its detailed operational procedure.

In the part of testing, a typical flight mission, with large variation ranges of signal power and dynamic stress, is taken as a destination object. The trajectory of the object is hybrid, which consists of a period of normal status (non-maneuvering) followed with a period of orbital maneuvering. The simulation results verify the fact that the performance of an adaptive KF-based strategy is superior to the classical CL structure, especially when the object operates in a non-maneuvering status. However, in orbital maneuvering conditions, an adaptive KF-based strategy is incompetent to perform well as what the INS-assisted strategy does. Additionally, the non-coherent MLE method in the INS-assisted strategy provides more robustness to track the Doppler frequency.

The remainder of the paper is organized as follows: Section 2 establishes a reference assumption PLL structure as the baseline of state-of-art; in Section 3, a four-state KF-based signal tracking method is presented which can adjust the equivalent LNBW adaptively; Section 4 depicts the architecture of the INS-assisted open loop design, and further proposes the non-coherent MLE algorithm applied in the architecture. Simulation experiments are carried out to test the effectiveness and feasibility of our strategies in Section 5; and finally, the paper is concluded by outlining the distinctive benefits in Section 6.

## 2. Reference Assumption PLL Features

Generally, GNSS applications suffer from some technical problems in harsh environments. For instance, indoor localization is trying to invent high-sensitivity receivers to receive weak signals, while missile guidance requires a better capacity to bear high-dynamics. As a result, the technologies aimed at solving high-sensitivity and high-dynamics are developed separately. However, space navigation with GNSS in the domain of SSV is influenced by the problem of realizing simultaneous high-sensitivity and high-dynamics, especially when the space vehicles are making orbital maneuvers. Theoretically, it is much more difficult to settle both of the two problems which interact with each other. To evaluate the performance of a current onboard GNSS receiver convincingly, we must establish a unified standard loop named SSV RRAM first.

### 2.1. The Definition of SSV RRAM

The so-called ‘dynamics’, in a generic sense, are concerned with velocity, acceleration, and jerk of the relative movement between the GNSS satellite and the user vehicle. Considering the motion complexity of SSV users, a space vehicle equipped with a third-order PLL is treated as the baseline of current technology. A third-order PLL is recommended for SSV RRAM resulting from its unique attributes. First, a third-order PLL can track the variations of velocity and acceleration without any bias, which is prior to first-order and second-order PLLs. Second, the jerk is so small for non-maneuvering space vehicles that the steady state dynamic stress error caused by jerk is also small. With regard to the parameter design of the SSV RRAM, two core parameters, the LNBW and CIT, should be emphatically analyzed.

The interaction effect of high-sensitivity and high-dynamics is reflected by these two parameters. Reducing the tracking loop bandwidth is the most effective way to achieve high-sensitivity, but a narrower LNBW is not in the best interest of high-dynamics. Secondly, increasing CIT is another way to achieve high-sensitivity, but the Doppler frequency changes dramatically in high-dynamic conditions, which results in an unacceptable integration loss caused by frequency misalignment. Apparently, neither reducing the LNBW nor increasing CIT is able to satisfy high-sensitivity and high-dynamic performance at the same time. The contradiction analyzed above brings about an issue about how to select proper LNBW and CIT for our SSV RRAM.

### 2.2. The Parameter Design of SSV RRAM

The carrier tracking loop measurement errors consist of two portions, phase jitter ($\sigma_{j}$), and line-of-sight (LOS) dynamic stress error $\theta_{e}$. The former is subdivided into three parts: the thermal noise $\sigma_{tPLL}$, the vibration-induced oscillator jitter, and the Allan variance-induced oscillator jitter. When a user is transferred from a normal condition into a weak-signal and high-dynamic environment, the thermal noise increases as the C/N_0_ decreases, while the dynamic stress error increases as the dynamics become greater. Apparently, for SSV users, the thermal noise and dynamic stress error are the two dominant PLL error sources. Both of them are far greater than the two kinds of oscillator jitters, so the influence of oscillator jitters can be ignored herein. Acting as the major constituent of phase jitter, the thermal noise is usually expressed in the following equation according to [12]:

(1) $$\sigma_{tPLL}=\frac{180}{\pi }\sqrt{\frac{B_{L}}{C/N_{0}}(1+\frac{1}{2T_{coh}\cdot C/N_{0}})}$$

where $B_{L}$ represents the LNBW, and $T_{coh}$ represents the CIT. It must be stressed that $C/N_{0}$ remains unchanged before and after coherent integration.

For the SSV RRAM, the steady state error caused by LOS dynamic stress is written as:

(2) $$\sigma_{d}=\frac{1}{\omega_{n}^{3}}\frac{d^{3}R}{dt^{3}}=\frac{0.7845^{3}}{B_{L}^{3}}J_{LOS}(m)=\frac{0.7845^{3}}{B_{L}^{3}}J_{LOS}\cdot \frac{360f_{L1}}{c}(\deg)$$

where *c* is the speed of light and $f_{L1}$ is the carrier frequency of L1 signal. $\frac{d^{3}R}{dt^{3}}$ is the third-order derivative of LOS range with respect to time, which is equivalent to $J_{LOS}$, i.e., the projection of jerk between navigation satellite and user vehicle in the direction of LOS. $\omega_{n}$ represents the natural frequency, and has a definite relationship with $B_{L}$ in the third-order PLL:

(3) $$\omega_{n}=\frac{B_{L}}{0.7845}$$

#### 2.2.1. The Determination of CIT

The length of the CIT is restricted to two factors, the length of navigation data bit and integration loss caused by frequency error. In weak-signal and high-dynamic environments, which of the two factors is dominant should be identified with quantitative calculation as follows. In order to keep the integration loss caused by frequency error $f_{e}$ below 3 dB [13], we have:

(4) $$\left|\sin c(f_{e}T_{coh})\right|\leq \frac{1}{\sqrt{2}}$$

Under high-dynamic circumstances, $f_{e}$ mainly comes from the relative movements between the navigation satellite and the user vehicle. Take $v_{LOS}$ and $a_{LOS}$ as projections of relative velocity and acceleration from the satellite to the vehicle in the *LOS* direction, respectively; the Doppler frequency is:

(5) $$f_{d}=-\frac{f_{L1}\cdot v_{LOS}}{c}$$

The rate of Doppler frequency ${\dot{f}}_{d}$ equals the time derivative of $f_{d}$:

(6) $${\dot{f}}_{d}=\frac{df_{d}}{dt}=-\frac{f_{L1}\cdot a_{LOS}}{c}$$

Furthermore, the rate of ${\dot{f}}_{d}$ can be expressed with $J_{LOS}$:

(7) $${\ddot{f}}_{d}=\frac{d^{2}f_{d}}{dt^{2}}=-\frac{f_{L1}\cdot J_{LOS}}{c}$$

Then the frequency error during the integration processing can be computed as:

(8) $$f_{e}=f_{d}+{\dot{f}}_{d}\cdot T_{coh}+\frac{1}{2}{\ddot{f}}_{d}T_{coh}^{2}=-\frac{f_{L1}\cdot v_{LOS}}{c}-\frac{f_{L1}\cdot a_{LOS}}{c}T_{coh}-\frac{f_{L1}\cdot J_{LOS}}{2c}T_{coh}^{2}$$

Assume that the first two terms of the right side of Equation (8), related to $v_{LOS}$ and $a_{LOS}$, respectively, is completely compensated by the third-order loop; then the equation is simplified as:

(9) $$f_{e}=-\frac{f_{L1}\cdot J_{LOS}}{2c}T_{coh}^{2}$$

Substituting Equation (9) into Equation (4), we have:

(10) $$\left|\sin c(-\frac{\pi f_{L1}J_{LOS}T_{coh}^{3}}{2c})\right|\leq \frac{1}{\sqrt{2}}$$

**Figure 1:**
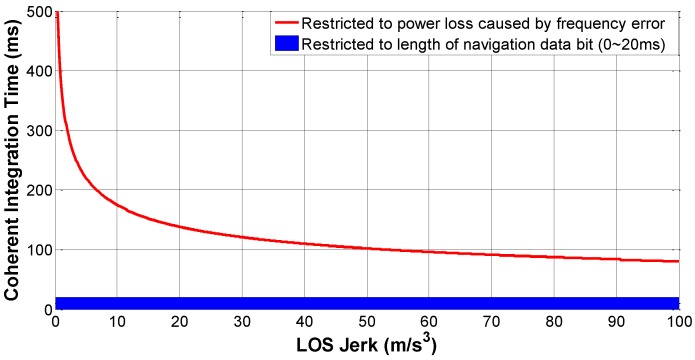
The selection of CIT over different LOS jerks.

Thus, the upper limitation of $T_{coh}$ is determined:

(11) $$T_{coh}=1000\left[\sqrt[{3}]{\frac{0.886c}{\pi f_{L1}J_{LOS}}}\right](ms)$$

where the operator [x] represents the maximum rounding operation below the value x, for the reason that $T_{coh}$ equals the integer multiples of C/A code period (1 ms). Figure 1 presents the bound of $T_{coh}$ over the variation of $J_{LOS}$, and proves that CIT restricted to the length of navigation data bit is dominant when $J_{LOS}$ does not exceed 100 m/s^3^. With regard to the mission in Section 5, whose peak jerk is 40 m/s^3^, a CIT of 20 ms would not lead to an unacceptable power loss.

#### 2.2.2. The Determination of LNBW

As discussed above, the errors from oscillator jitters are ignored in SSV applications, and the rule-of-thumb expression of the loop measurement error is expressed as the following equation [13]:

(12) $$\sigma_{PLL}=\sigma_{tPLL}+\frac{1}{3}\sigma_{d}=\frac{180}{\pi }\sqrt{\frac{B_{L}}{C/N_{0}}(1+\frac{1}{2T_{coh}C/N_{0}})}+\frac{57.94f_{L1}J_{LOS}}{cB_{L}^{3}}$$

The SSV RRAM chooses a two-quadrant arctangent discriminator, which is the most accurate one among all kinds of phase detectors. Referring to prior discoveries [12], the one-sigma rule threshold for the two-quadrant arctangent discriminator is 15°. Then we get the maximum bearable jerk of the third-order PLL over the variation of $B_{L}$:

(13) $$J_{LOS}=\frac{\left[15^{\circ }-\frac{180}{\pi }\sqrt{\frac{B_{L}}{C/N_{0}}(1+\frac{1}{2T_{coh}C/N_{0}})}\right]cB_{L}^{3}}{57.94f_{L1}}$$

**Figure 2:**
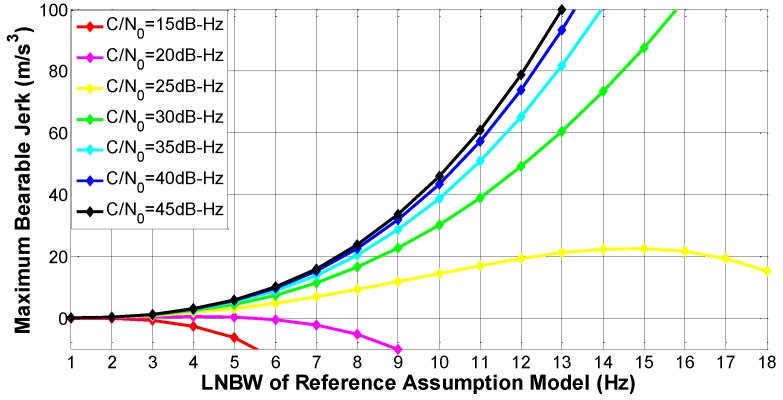
The maximum bearable LOS jerk under different C/N_0_ for SSV RRAM.

In the case that the details of data bit transition are unknown, we set the CIT at 20 ms. Considering the stable condition of the third-order loop is $B_{L}\cdot T_{coh}<1.0$ [14], $B_{L}$ changes from 1 Hz to 18 Hz. According to [4], the minimum received C/N_0_ is about 14.59 dB-Hz for SSV users. Meanwhile, the normal C/N_0_ is about 45 dB-Hz for ground users [13]. Thus, we choose the C/N_0_ values ranging from 15 dB-Hz to 45 dB-Hz for weak signal analysis. Then we draw the fluctuation of the maximum bearable jerk in different power levels in Figure 2, where seven different C/N_0_ values are selected from 15 dB-Hz to 45 dB-Hz with a uniform spacing of 5 dB-Hz. Without a doubt, a negative jerk is physically meaningless, but the negative jerks in Figure 2 represent the PLL is certainly out of lock under these conditions. The figure shows two important facts:

1. The third-order PLL is vulnerable to bear any dynamics when the power becomes very weak (20 dB-Hz or lower);
2. The threshold of maximum bearable jerk increases with the growth of LNBW when the received C/N_0_ is greater than 30 dB-Hz, but vibrates under a weak signal environment. Thus, there is an optimal LNBW that minimizes the total loop measurement error.

From the two facts, we can conclude that our SSV RRAM is incompetent to realize both high-sensitivity and high-dynamics simultaneously.

## 3. Adaptive Bandwidth Tracking Strategy

Based on the second fact in the end of last section, this section introduces the optimization of $B_{L}$. To minimize the thermal noise error, a narrower $B_{L}$ is desirable. To minimize the LOS dynamic stress error, a wider $B_{L}$ is necessary. Obviously, $B_{L}$ should be optimized to bring down the weighted sum of thermal noise and dynamic stress error. The theoretical optimum LNBW and the method to get the optimal solution are introduced in this section.

### 3.1. Theoretical Optimum LNBW

Computing the partial derivative of $\sigma_{PLL}$ to $B_{L}$, we get $\frac{\partial \sigma_{PLL}}{\partial B_{L}}$. When $\frac{\partial \sigma_{PLL}}{\partial B_{L}}=0$, the value of $B_{L}$ that make the $\sigma_{PLL}$ minimal can be determined:

(14) $$B_{L\_optimal}=\sqrt[{7}]{\frac{173.8^{2}\pi^{2}J_{LOS}^{2}f_{L1}^{2}}{90^{2}c^{2}}\cdot \frac{2T_{coh}{\left(C/N_{0}\right)}^{2}}{2T_{coh}\cdot C/N_{0}+1}}$$

In practical SSV flight missions, both the C/N_0_ and $J_{LOS}$ are changing continuously. To achieve an optimal $B_{L}$, a self-adaptive method should be utilized, and the adaptive KF-based algorithm is exactly such a method.

### 3.2. Adaptive Four-State KF-Based Algorithm

**Figure 3:**
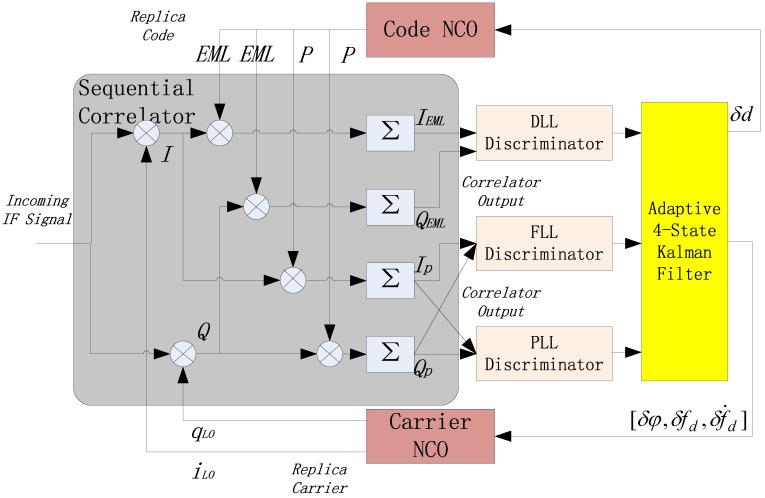
Block diagrams of adaptive four-state KF-based tracking strategy.

A three-state (phase error, Doppler frequency, and Doppler frequency rate) adaptive KF-based method was adopted in [15]. To improve the loop performance in weak-signal and high-dynamic environments, we introduce the state of code phase error and extend the three-state to four-state. Replacing DLL (Delay Lock Loop), FLL (Frequency Lock Loop), and PLL filters of conventional tracking structure with a combined adaptive Kalman filter, the block diagram of the four-state KF-based strategy is depicted in Figure 3. The error-state vector consists of code phase error $\delta d_{k}$, carrier phase error $\delta \phi_{k}$, Doppler frequency error $\delta f_{d,k}$, and its rate error $\delta {\dot{f}}_{d,k}$ at time k:

(15) $$X_{k}={\left[\delta d_{k},\delta \phi_{k},\delta f_{d,k},\delta {\dot{f}}_{d,k}\right]}^{T}$$

$\delta f_{d,k}$ equals the difference between the actual Doppler frequency and the frequency feedback to numerically controlled oscillator(NCO). For L1 C/A signal, the ratio of code chip rate $f_{code}=1.023\;\mathrm{Mcps}$ and nominal radio frequency $f_{RF}=1575.42\;\mathrm{MHz}$ is constant, so the state forward prediction is expressed as:

(16) $${\hat{X}}_{k,k-1}=\Phi_{k,k-1}{\hat{X}}_{k-1}=\left[\begin{array}{cccc} 1 & 0 & \frac{f_{code}T_{coh}}{f_{RF}} & \frac{f_{code}T_{coh}^{2}}{2f_{RF}}\\ & 1 & 2\pi T_{coh} & \frac{f_{code}T_{coh}^{2}}{2f_{RF}}\\ & & 2\pi & 2\pi T_{coh}\\ & & & 2\pi \end{array}\right]{\hat{X}}_{k-1}=\left[\begin{array}{cccc} 1 & 0 & \frac{T_{coh}}{1540} & \frac{T_{coh}^{2}}{2\cdot 1540}\\ & 1 & 2\pi T_{coh} & \frac{T_{coh}^{2}}{2\cdot 1540}\\ & & 2\pi & 2\pi T_{coh}\\ & & & 2\pi \end{array}\right]{\hat{X}}_{k-1}$$

Since coherent integration is used to process weak signals, the measurements obtained from discriminators are the average phase differences during CIT, rather than instant phase errors. Thus, we have:

(17) $$\delta \phi_{k}=\frac{1}{T_{coh}}\int_{0}^{T_{coh}}\delta \phi_{k-1}+2\pi \delta f_{d,k-1}\tau +\frac{1}{2}2\pi \delta {\dot{f}}_{d,k-1}\tau^{2}d\tau =\delta \phi_{k-1}+\pi \delta f_{d,k-1}T_{coh}+\frac{1}{3}\pi \delta {\dot{f}}_{d,k-1}T_{coh}^{2}\;=\left[\begin{array}{cccc} 0 & 1 & \pi T_{coh} & \frac{1}{3}\pi T_{coh}^{2} \end{array}\right]X_{k-1}$$

Likely, the measurement equation of code phase error is:

(18) $$\delta d_{k}=\left[\begin{array}{cccc} 1 & 0 & \frac{\pi T_{coh}}{1540} & \frac{\pi T_{coh}^{2}}{3\cdot 1540} \end{array}\right]X_{k-1}$$

Rewriting Equations (17) and (18) in the form of matrix and get the measurement matrix:

(19) $$H_{k}=\left[\begin{array}{cccc} 1 & 0 & \frac{\pi T_{coh}}{1540} & \frac{\pi T_{coh}^{2}}{3\cdot 1540}\\ \;0 & \;1 & \;\pi T_{coh} & \frac{1}{3}\pi T_{coh}^{2} \end{array}\right]$$

The formal measurement equation is expressed as:

(20) $$Z_{k}=H_{k}\Phi_{k,k-1}X_{k-1}+v_{k}$$

where $v_{k}$ stands for the difference between actual measurement obtained from discriminator and the theoretical forward prediction. The process noise matrix is computed as follows:

(21) $$Q_{k}=E[({\hat{X}}_{k}-{\hat{X}}_{k-1}){\left({\hat{X}}_{k}-{\hat{X}}_{k-1}\right)}^{T}]\;=S_{f}f_{L1}^{2}\left[\begin{array}{cccc} \frac{T_{coh}}{1540^{2}} & \frac{T_{coh}}{1540} & 0 & 0\\ \frac{T_{coh}}{1540} & T_{coh} & 0 & 0\\ 0 & 0 & 0 & 0\\ 0 & 0 & 0 & 0 \end{array}\right]+S_{g}f_{L1}^{2}\left[\begin{array}{cccc} \frac{T_{coh}^{3}}{3\cdot 1540^{2}} & \frac{T_{coh}^{3}}{3\cdot 1540} & \frac{T_{coh}^{2}}{2\cdot 1540} & 0\\ \frac{T_{coh}^{3}}{3\cdot 1540} & \frac{T_{coh}^{3}}{3} & \frac{T_{coh}^{2}}{2} & 0\\ \frac{T_{coh}^{2}}{2\cdot 1540} & \frac{T_{coh}^{2}}{2} & T_{coh} & 0\\ 0 & 0 & 0 & 0 \end{array}\right]\;+q_{a}{\left(\frac{f_{L1}}{c}\right)}^{2}\left[\begin{array}{cccc} \frac{T_{coh}^{5}}{20\cdot 1540^{2}} & \frac{T_{coh}^{5}}{20\cdot 1540} & \frac{T_{coh}^{4}}{8\cdot 1540} & \frac{T_{coh}^{3}}{6\cdot 1540}\\ \frac{T_{coh}^{5}}{20\cdot 1540} & \frac{T_{coh}^{5}}{20} & \frac{T_{coh}^{4}}{8} & \frac{T_{coh}^{3}}{6}\\ \frac{T_{coh}^{4}}{8\cdot 1540} & \frac{T_{coh}^{4}}{8} & \frac{T_{coh}^{3}}{3} & \frac{T_{coh}^{2}}{2}\\ \frac{T_{coh}^{3}}{6\cdot 1540} & \frac{T_{coh}^{3}}{6} & \frac{T_{coh}^{2}}{2} & T_{coh} \end{array}\right]+q_{d}\left[\begin{array}{cccc} T_{coh} & 0 & 0 & 0\\ 0 & 0 & 0 & 0\\ 0 & 0 & 0 & 0\\ 0 & 0 & 0 & 0 \end{array}\right]$$

where $q_{a}$ is the spectral density of LOS acceleration random walk model, $q_{d}$ is the code density of LOS acceleration random walk model, $S_{f}$ and $S_{g}$ are relevant to the phase and frequency of clock oscillator. Generally, $S_{f}=2h_{0}$ and $S_{g}=8\pi^{2}h_{-2}$. The coefficients $h_{0}$ and $h_{-2}$ are determined by the type of clock oscillator [16,17], which can be referred in Table 1.

Additionally, the measurement noise covariance matrix is related to the output of the discriminator, and it is a function of real-time signal power:

(22) $$R_{k}=\left[\begin{array}{cc} \sigma_{d}^{2} & \\ & \sigma_{\phi }^{2} \end{array}\right]=\left[\begin{array}{cc} d(2-d){\left[\frac{1}{2}+\frac{1}{T_{coh}C/N_{0}(2-d)}\right]}^{2} & \\ & \frac{1}{2T_{coh}C/N_{0}}(1+\frac{1}{2T_{coh}C/N_{0}})I \end{array}\right]$$

where d is the code phase, $\sigma_{d}^{2}$ is noise variance of the DLL discriminator, $\sigma_{\phi }^{2}$ is noise variance of the PLL discriminator, and I represents the unit diagonal matrix. The iterative process of KF contains the computation of forward prediction covariance matrix $P_{k,k-1}$, adaptive gain matrix $K_{k}$, new state covariance matrix $P_{k}$ and new state vector $X_{k}$:

(23) $$\{\begin{array}{c} P_{k,k-1}=\Phi_{k,k-1}P_{k-1}\Phi_{k,k-1}^{T}+Q_{k}\\ K_{k}=P_{k,k-1}H_{k}^{T}{\left[R_{k}+H_{k}P_{k,k-1}H_{k}^{T}\right]}^{-1}\\ P_{k}=(I-K_{k}H_{k})P_{k,k-1}\\ {\hat{X}}_{k}={\hat{X}}_{k,k-1}+K_{k}v_{k} \end{array}$$

Compared to conventional tracking strategy, the advantage of an adaptive KF is that the equivalent LNBW can be easily adjusted by tuning the coefficients in the noise matrix. In-depth analysis was given by [18] about how the adaptive KF varies the LNBW in a time-varying optimal manner as the signal power and dynamics change. The equivalent LNBW in steady state is:

(24) $${\left(B_{L\_eq}\right)}_{k}≅\frac{K_{k}(Q_{k},R_{k})}{c_{3}T_{coh}}=\frac{(\Phi_{k,k-1}P_{k-1}\Phi_{k,k-1}^{T}+Q_{k})H_{k}^{T}{\left[R_{k}+H_{k}(\Phi_{k,k-1}P_{k-1}\Phi_{k,k-1}^{T}+Q_{k})H_{k}^{T}\right]}^{-1}}{c_{3}T_{coh}}$$

where the filter coefficient $c_{3}$ for the third-order PLL equals 3.048 and the gain matrix $K_{k}$ is a function of $Q_{k}$ and $R_{k}$. It can be proved than $K_{k}$ is positively correlated with $Q_{k}$ and negatively correlated with $R_{k}$, thus $K_{k}\propto \frac{Q_{k}}{R_{k}}$. This indicates the realization of adaptive bandwidth in weak-signal and high dynamic environment:

1. When the signal turns weaker, the C/N_0_ becomes lower, so that the measurement noise $R_{k}$ becomes greater, $K_{k}$ decreases further and, finally, the LNBW is narrowed;
2. When the LOS dynamic increases, the process noise $Q_{k}$ becomes greater, $K_{k}$ increases further and, finally, the LNBW is widened.

## 4. INS-Assisted Open Loop Tracking Strategy

As an opposite method against the closed loop, the idea of the open loop has been discussed for many years. In our common sense, open loop tracking is considered as block processing or batch processing. Contrary to closed loop sequential processing, the open loop batch processing has no local loop updates [19]. The use of open loop batch processing allows parallel computations on the batches of samples, and brings the benefits of improved signal observability and improved tracking robustness [20]. This section illustrates the structure of INS-assisted tracking strategy and the frequency estimation algorithm adopted in this strategy.

### 4.1. Structure of INS-Assisted Tracking Strategy

**Figure 4:**
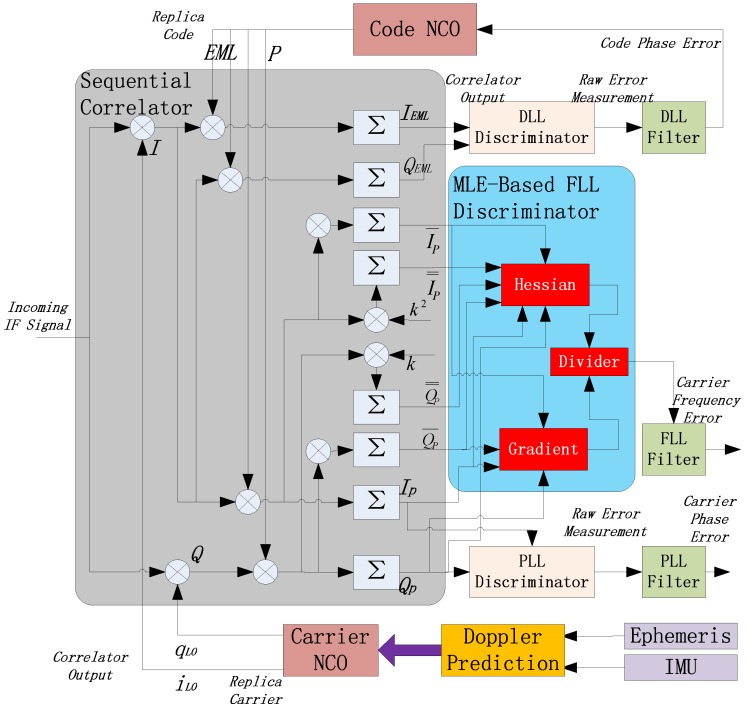
Suggested architecture of INS-assisted open loop tracking.

It must be admitted that OL batch processing is less favorable than the CL sequential method with respect to computation efficiency, resulting from the complicated FFT calculation. Additionally, the OL batch scheme does not improve the measurement accuracy as compared to the CL sequential architecture. Instead, the OL scheme mainly promotes the robustness and reliability of the tracking loop, especially in challenging environment, such as SSV applications. Thus, it is necessary to put forward a new OL structure to ease the computation burden. In Figure 4, a suggested OL architecture is proposed with this purpose.

The proposed architecture is built on the basis of Doppler prediction utilizing inertial measurement unit (IMU) and GNSS ephemeris. IMU is a cluster of sensors, including accelerometers and gyros, which is responsible for obtaining the motion and attitude of the SSV user receiver, whereas the GNSS satellite ephemeris is used to calculate the position and velocity of the satellite by accurate orbit propagation models. Referring to [21], the Doppler frequency shift caused by the motion of the SSV user receiver relative to the GNSS satellite can be easily determined. The estimation of the Doppler frequency is used to reduce the local frequency search space, and the estimation accuracy depends on the quality of IMU. If the space vehicles are equipped with high quality IMUs, the expected Doppler frequency error accumulates slowly over time, which is adequate for SSV navigation. The ephemeris is obtained during visible intervals and saved into onboard registers for orbital propagation. It has been proved that the outage time when no satellite is in view does not exceed 108 min for SSV flight missions [1], and the length of the orbit propagation is relatively short so that the dynamic aiding is accurate enough.

After A/D (Analog-to-Digital) conversion, the digitized incoming IF signal is generally expressed as:

(25) $$s(k)=AC(k-\tau )D(k)\cos[2\pi kT(f_{IF}+f_{d})+\varphi ]+n(k)$$

where k is the time index of each sampling time interval, and T is the sampling time interval; A is the signal amplitude, C(⋅) is the spreading code sequence and D(⋅) is the navigation data bit; the three parameters to be estimated are code delay τ, Doppler frequency shift $f_{d}$, and carrier phase φ; $f_{IF}$ is IF in Hz, and n(k) is additive band-limited white Gaussian noise (AWGN).

Using the dynamic aiding with IMU and ephemeris described in Figure 4, we get the estimated Doppler frequency ${\hat{f}}_{d}$. If the estimated initial phase is ${\hat{\phi }}_{0}$, the two orthogonal carriers representing the outputs of carrier NCO are:

(26) $$\{\begin{array}{c} i_{LO}(k)=\cos[2\pi (f_{IF}+{\hat{f}}_{d})kT+{\hat{\phi }}_{0}]\\ q_{LO}(k)=\sin[2\pi (f_{IF}+{\hat{f}}_{d})kT+{\hat{\phi }}_{0}] \end{array}$$

The received signals in Equation (25) are correlated with the local replica signals in Equation (26) to generate the correlated results as:

(27) $$\{\begin{array}{c} I(k)=AR(\delta \tau )D(k)\cos(2\pi kT\cdot \delta f+\delta \phi_{0})+n_{i}(kT)\\ Q(k)=AR(\delta \tau )D(k)\sin(2\pi kT\cdot \delta f+\delta \phi_{0})+n_{q}(kT) \end{array}$$

where δf is the Doppler frequency estimation error, $\delta \phi_{0}$ is the initial phase estimation error, δτ is the code delay error, R(⋅) is the autocorrelation function (ACF) of C/A code, $n_{i}$ is the in-phase noise and $n_{q}$ is the quadrature noise. After coherent integration, we get the integration results of $I_{P}$ and $Q_{P}$ [22]:

(28) $$\{\begin{array}{l} I_{P}(k)=AR(\delta \tau )D(k)\sin c(kT\cdot \delta f)\cos[2\pi \delta f(kT+\frac{T_{coh}}{2})+\delta \phi_{0}]\\ Q_{P}(k)=AR(\delta \tau )D(k)\sin c(kT\cdot \delta f)\sin[2\pi \delta f(kT+\frac{T_{coh}}{2})+\delta \phi_{0}] \end{array}$$

It is obvious that the estimation of $\delta \phi_{0}$ and δf are tightly coupled. The PLL discriminator output $\delta \phi_{0}$ is influenced by δf, while the FLL discriminator output δf is influenced by $\delta \phi_{0}$. The calculation results obtained directly from $I_{P}$ and $Q_{P}$ in Equation (28) are unreliable, so a non-coherent MLE-based FLL discriminator is applied in the architecture shown in Figure 4 to settle the problem.

### 4.2. Non-Coherent MLE Algorithm

Hereinafter, a non-coherent MLE algorithm to estimate Doppler frequency error that is insensitive to carrier phase error is introduced. By computing the sum of the squares of $I_{P}$ and $Q_{P}$, we have the value of non-coherent integration (NI):

(29) $$V=I_{P}^{2}(k)+Q_{P}^{2}(k)=A^{2}{\left[R(\delta \tau )\right]}^{2}{\left[D(k)\right]}^{2}{\left[\sin c(kT\cdot \delta f)\right]}^{2}\;=A^{2}{\left[R(\delta \tau )\right]}^{2}{\left[\sin c(kT\cdot \delta f)\right]}^{2}$$

It is obvious that NI brings about two positive features: First, the value of V is not affected by data bit D(k) regardless of whether D(k) equals +1 or −1, so the influence of data bit transition is eliminated; Second, the value of V has nothing to do with carrier phase error $\delta \phi_{0}$, and it changes with δf. The two features induce us to construct a maximum likelihood cost function for Doppler estimation using the NI result, and the expression is as follows:

(30) $$L\left(\delta f\right)=\sum_{k=1}^{N}\left[I_{P}^{2}(k)+Q_{P}^{2}(k)\right]=\sum_{k=1}^{N}A^{2}{\left[R(\delta \tau )\right]}^{2}{\left[\sin c(kT\cdot \delta f)\right]}^{2}$$

where N represents the total number of IF data samples within a given CIT. According to the normal MLE solving method, δf reaches its MLE where the partial derivative of the likelihood cost function or its log-likelihood function [23] with respect to δf is 0. However, obviously, it is too complicated to write the analytic expression of the MLE of δf. Thus, we rewrite the cost Function (30) as:

(31) $$L\left(\delta f\right)=\sum_{k=1}^{N}\left[I_{P}^{2}(k)+Q_{P}^{2}(k)\right]=\sum_{k=1}^{N}{\left[s(k)\cdot C\cdot \cos\right]}^{2}+\sum_{k=1}^{N}{\left[s(k)\cdot C\cdot \sin\right]}^{2}$$

For the sake of simplicity, we use C as C(k−τ), cos as $\cos[2\pi (f_{IF}+\delta f)kT+\delta \phi ]$, and sin as $\sin[2\pi (f_{IF}+\delta f)kT+\delta \phi ]$. Then the gradient of the new cost function for the Doppler frequency is given by:

(32) $$Gradient=\frac{\partial L}{\partial \delta f}=-4\pi T\sum_{k=1}^{N}[s(k)\cdot C\cdot \cos]\times \sum_{k=1}^{N}\left[k\cdot s(k)\cdot C\cdot \sin\right]\;+4\pi T\sum_{k=1}^{N}[s(k)\cdot C\cdot \sin]\times \sum_{k=1}^{N}\left[k\cdot s(k)\cdot C\cdot \cos\right]=-4\pi T(I_{P}{\bar{Q}}_{P}-{\bar{I}}_{P}Q_{P})$$

where:

(33) $$\{\begin{array}{c} I_{P}=\sum_{k=1}^{N}s(k)\cdot C\cdot \cos\\ Q_{P}=\sum_{k=1}^{N}s(k)\cdot C\cdot \sin\\ {\bar{I}}_{P}=\sum_{k=1}^{N}k\cdot s(k)\cdot C\cdot \cos\\ {\bar{Q}}_{P}=\sum_{k=1}^{N}k\cdot s(k)\cdot C\cdot sin \end{array}$$

The Hessian [24] of the proposed cost function with regard to δf is given by:

(34) $$Hessian=\frac{\partial^{2}L}{\partial \delta f^{2}}=8\pi^{2}T^{2}({\bar{I}}_{P}^{2}+{\bar{Q}}_{P}^{2}-I_{P}{\overset{=}{I}}_{P}-Q_{P}{\overset{=}{Q}}_{P})$$

where:

(35) $$\{\begin{array}{c} {\overset{=}{I}}_{P}=\sum_{k=1}^{N}k^{2}\cdot s(k)\cdot C\cdot \cos\\ {\overset{=}{I}}_{P}=\sum_{k=1}^{N}k^{2}\cdot s(k)\cdot C\cdot sin \end{array}$$

Referring to [25], the frequency error is estimated with the gradient divided by the Hessian:

(36) $$\delta f=-\frac{Gradient}{Hessian}=\frac{I_{P}{\bar{Q}}_{P}-{\bar{I}}_{P}Q_{P}}{2\pi T({\bar{I}}_{P}^{2}+{\bar{Q}}_{P}^{2}-I_{P}{\overset{=}{I}}_{P}-Q_{P}{\overset{=}{Q}}_{P})}$$

The calculation is completed in the block named MLE-based FLL discriminator in Figure 4. Finally, the coupling effect between Doppler frequency error and carrier phase error is cancelled.

In addition, the code delay τ is still estimated with CL scheme using a carrier-aided DLL. There are two early-minus-late (EML) type correlators with outputs:

(37) $$\{\begin{array}{l} I_{EML}=\sum_{k=1}^{N}s(k)\cdot \dot{C}\cdot \cos\\ Q_{EML}=\sum_{k=1}^{N}s(k)\cdot \dot{C}\cdot sin \end{array}$$

where the new symbol $\dot{C}$ is the derivative of C with respect to τ. For a definite correlator spacing d, it is calculated with the following equation:

(38) $$\dot{C}=\frac{\partial C(k-\tau )}{\partial \tau }\approx \frac{C(k-\tau -\frac{d}{2})-C(k-\tau +\frac{d}{2})}{d}$$

## 5. Simulation and Experiment Results

Under normal conditions, the comparison between loop measurement error and its threshold is used to judge whether the loop is out of lock. Considering the balance of both measurement accuracy and rapid response capability, a threshold of 15° is regarded as a judgment indicator about whether the loop is out of lock or not in this section. Hereafter, a testing scenario is built up at first, then the experimental system and initial settings are introduced, and the Doppler frequency estimation error is used to weigh the performance of our tracking strategies.

### 5.1. Scenario Settings

**Figure 5:**
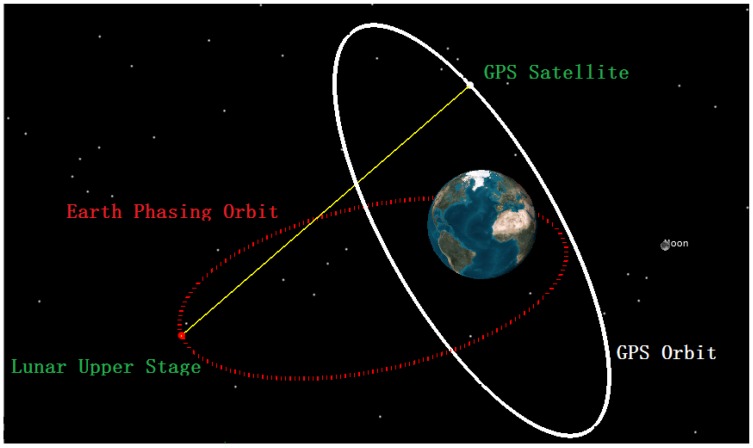
Schematic diagram of the simulation scenario.

In order to identify the tracking quality of our proposed strategies, we establish a scenario of a lunar upper stage on a HwaCreat™ GNSS signal simulator (produced by Beijing Hwa Creat Technology Corporation Ltd., Beijing, China). In the scenario, the lunar exploration probe operates in its Earth phasing orbit [4], whose trajectory traverses both TSV and SSV in a geostationary transfer orbit (GTO). One certain GPS satellite is chosen as the signal source, and the signal emitted from the satellite is used for analysis when it is visible to our object vehicle. The simulated scenario is drawn in Figure 5. We can see the lunar upper stage receives the signal emitted by the GPS satellite and transmitted over the limb of the Earth, which is consistent with the basic characterization of SSV navigation. The simulation time lasts for 320 s, which is divided into four phases. During the simulation time, the lunar probe operates at an altitude of about 35,000 km, and transfers from its original phasing orbit to another phasing orbit by means of an orbital maneuver. In the first 50 s, the GPS satellite is not visible to the lunar probe. From the 51st s to 300th s, the GPS satellite enters the visible area of the lunar probe. In this period, the probe operates under gravity, and completely free from its own drag force. In the third phase, from the 301st s to the 310th s, the onboard motor starts to work and generates a ramp LOS jerk. The value of the LOS jerk reaches its maximum at the 310th s and keeps constant in the fourth phase. The dynamic performance requirements for the GNSS receiver of the lunar upper stage can be seen in Table 2. If the Doppler shift caused by the LOS velocity and acceleration are completely compensated by the third-order PLL, the bearable jerk of 4 g/s becomes the most important requirement. Thus, we set the maximal LOS jerk at 40 m/s^3^ with a little excess. The mission control sequence (MCS) of the scenario is shown in Table 3.

**Figure 6:**
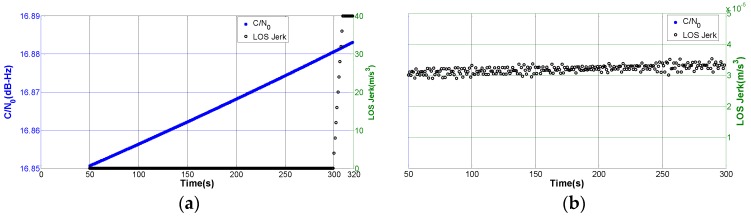
The variation of C/N_0_ and LOS jerk for the established scenario. (**a**) Global view; and (**b**) close-up view of the second phase.

Seen from Figure 6a, the received C/N_0_ and LOS jerk drawn in different colors are both changing over time. In the second phase, the LOS jerks are about 2.9~3.6 × 10^−5^ m/s^3^, which are too minor to be plotted clearly in the global view. Thus, we repaint the close-up view of LOS jerk from the 50th to 300th s in Figure 6b.

### 5.2. Experimental System and Initialization

**Figure 7:**
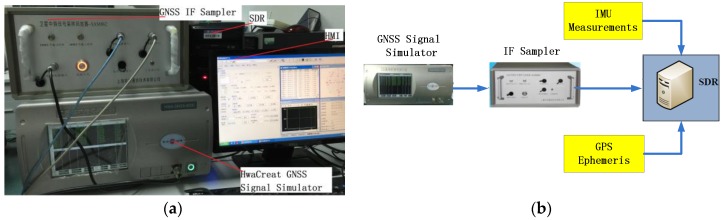
The distribution and connection of the instruments in the experiment. (**a**) Physical connection; and (**b**) schematic connection.

This subsection puts emphasis on the introduction of the experimental equipment being applied and their initialization settings. There are three instruments in the testing system: a HwaCreat™ GNSS signal simulator, an IF sampler, as well as a SDR (software-defined receiver). Their connection is shown in Figure 7. As aforementioned, we build up the scenario containing the lunar upper stage and a certain GPS satellite in the GNSS signal simulator. The radio frequency (RF) export of the simulator is connected to the IF sampler, so that the simulated signals can be collected and stored in the sampler. The sampler is capable of playback to ensure that the recorded data can be used for testing repeatedly, and make it possible that sufficient data can be obtained for statistical analysis. Finally, the digitized IF data is delivered into the SDR for processing. Note that the SDR is designed as three different structures in the following order: SSV RRAM, adaptive four-state KF-based structure illustrated in Figure 3, and INS-assisted structure illustrated in Figure 4.

For the conventional RRAM of CL form, the bandwidth of DLL, FLL, and PLL are 0.1 Hz, 2 Hz, and 18 Hz, respectively. The IF of the SDR is set at 4.092 MHz, whereas the sampling frequency is 16.368 MHz.

For the adaptive four-state KF-based strategy of CL form, the initial covariance matrix $P_{0}=diag(P_{00},P_{11},P_{22},P_{33})$, where $P_{00}=1chip^{2},P_{11}=1,P_{22}={\left(50Hz\right)}^{2},P_{33}={\left(1Hz/s\right)}^{2}$, and the initial state $X_{0}={\left(0,0,0,0\right)}^{T}$. Note that the values of the initialization can only affect the convergence speed of the filter instead of the estimation accuracy.

For the INS-assisted strategy of the OL form, the local Doppler frequency is predicted with the help of the input IMU measurements and GPS ephemeris. There are four different grade IMUs being used in the experiments: MEMS (micro electro mechanical system) grade, civil grade, tactical grade, and navigation grade. Their parameters are provided in Table 4. If the initial misalignment error is $\delta a_{0}$, the cumulative acceleration error can be modeled as a simplified form:

(39) $$\delta a_{LOS}=(\delta a_{0}+\nabla +g\cdot \varepsilon \cdot \Delta t)\cos\theta$$

where ∇ is the accelerometer error, ε is the gyro error and Δt is the drift time after the latest IMU calibration [26], and θ is the real-time projection angle between the IMU measurement vector and the LOS direction. In the case when the IMU correction to prevent drift error is unavailable, the estimated frequency error over drift time is:

(40) $$\delta f_{d}=-\frac{f_{L1}}{c}\int_{0}^{\Delta t}\left(\delta a_{0}+\nabla +g\cdot \varepsilon \cdot \tau \right)\cos\theta (\tau )d\tau$$

### 5.3. Result Comparison

**Figure 8:**
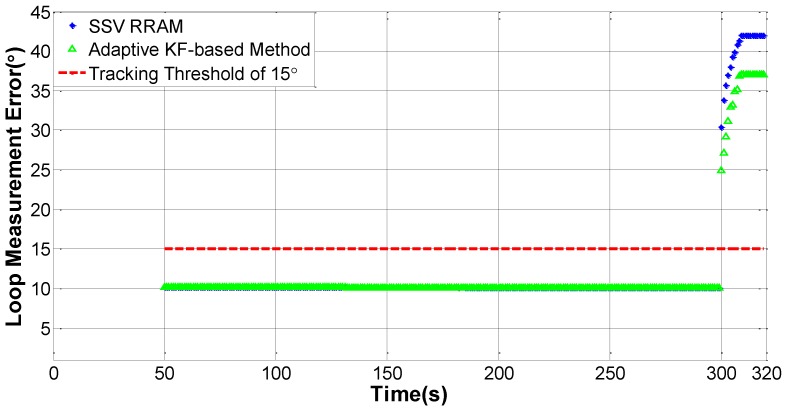
PLL lock status of two CL form tracking strategies, SSV RRAM and the adaptive KF-based method.

Seen from Figure 8, during the second phase, the loop measurement error obtained by an adaptive KF-based structure is roughly the same as that obtained by the conventional structure, namely SSV RRAM. However, in the third and fourth phases, the adaptive KF makes some improvement to reduce the measurement error, compared to SSV RRAM. It is clearly shown that the measurement errors of both the two structures exceed the tracking threshold of 15° after the 300th s. The result reveals several conclusions. First, the two schemes of CL form can work properly in non-maneuvering status when the C/N_0_ is over 16.85 dB-Hz. Second, adaptive KF performs better than SSV RRAM due to its flexibility of regulating LNBW in harsh environment. Third, both the two structures lose their locks in orbital maneuvering status and they are incapable of accomplishing navigation in the orbital transfer period of the lunar upper stage.

**Figure 9:**
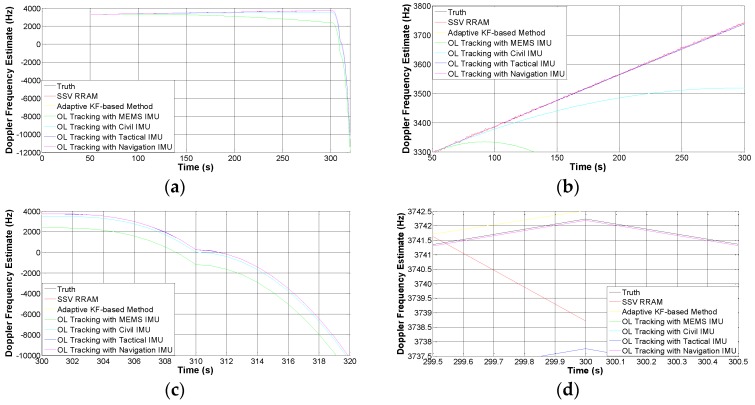
Comparison of the estimated Doppler frequency using different tracking schemes in the experiments. (**a**) Global view; (**b**) viewport of the second phase (non-maneuvering); (**c**) viewport of the third and fourth phase (maneuvering); and (**d**) close-up view around the 300th s.

Figure 9a shows a comparison of the Doppler frequency estimations of different tracking schemes in the same experiment scenario. The results are obtained through repeated trials for each scheme. In Figure 9b, no maneuver is made, all of the six schemes keep track of the target signal but the estimation accuracies diverge from each other. In Figure 9c, the probe makes an orbital maneuver, then the two CL schemes, SSV RRAM, as well as the adaptive KF-based method, are unavailable because the loop is out of lock. To demonstrate the performances of all these schemes clearly, we zoom in the plot around the 300th s with higher resolution in Figure 9d, and it is obvious that the estimation accuracy of INS-assisted strategy is determined by the quality of the IMUs. The IMU of the navigation grade is the best one, the tactical grade is secondary, the civil grade is tertiary, and the MEMS grade is the worst.

**Figure 10:**
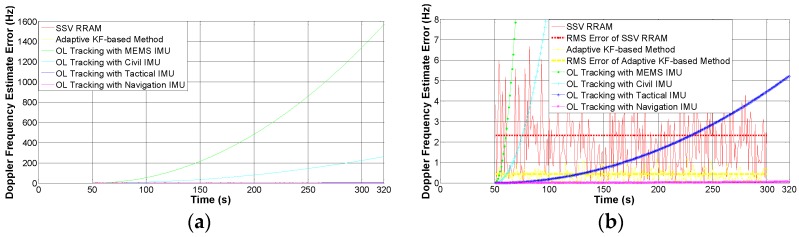
Comparison of Doppler estimation errors using different tracking schemes in the experiments. (**a**) Global view; and (**b**) close-up view.

Figure 10 provides the comparison of the estimation error of the six schemes. Through statistical computing of the estimation errors in the second phase, the root mean square (RMS) is 2.3188 Hz for SSV RRAM and 0.4324 Hz for the adaptive KF-based method. This leads to the conclusion that the adaptive KF-based method can get more precise estimates of the Doppler frequency than the conventional SSV RRAM when the space vehicle operates under normal or non-maneuvering condition. For the INS-assisted schemes of OL form, the frequency errors accumulate continuously. The maximum frequency errors are shown in Table 5 after a drift time of 270 s. It is obvious that the cumulative errors of the schemes aided by MEMS IMU and civil IMU grow quickly, which does not meet the performance requirements of SSV navigation. If the drift time without IMU calibration is short enough, e.g., within 77 s, the scheme aided by tactical IMU performs a little better than the adaptive KF-based method. However, once the drift time is over 78 s, the estimation error using the tactical IMU is above 0.4324 Hz which is inferior to the adaptive KF-based method. Although the scheme aided by navigation IMU is more accurate, the estimation error would exceed 0.4324 Hz after a cumulative time of 775 s. Therefore, if the drift time is too long or the dynamic is not too great, the adaptive KF-based method has its specific advantage compared to INS-assisted schemes. Therefore, what the OL tracking strategy actually improves is the loop robustness of GNSS signals tracking function, owing to the fact that OL tracking with high-quality IMU can work properly under orbital maneuvering conditions.

## 6. Conclusions

This section summarizes the contributions of this paper as follows. We first interpret why it is difficult to achieve high-sensitivity and high-dynamic GNSS signal tracking at the same time. Then a conventional design of a third-order PLL is put forward, and taken as the baseline, i.e., SSV RRAM. Taking both the low C/N_0_ and high LOS jerk into account, an optimized strategy of CL form is recommended, and this strategy can be implemented by an adaptive four-state KF-based algorithm, which can adjust its LNBW according to the received signal power and LOS dynamics. The paper also describes the relationship between the filter gain matrix and equivalent LNBW. In order to prevent the PLL from losing lock, an INS-assisted strategy is adopted, and the decoupling of the Doppler frequency estimate and carrier phase estimate should be attributed to the non-coherent processing. Finally, a mission of a lunar upper stage in its Earth phasing orbit is used to test the validity of these strategies. The simulation results substantially prove the superiority of an adaptive four-state KF-based strategy under non-maneuvering conditions and the advantage of an OL tracking strategy aided by high-quality IMUs under maneuvering conditions.

It must be noted that the analyses in the paper are all based on the steady state performance of the tracking structure, but the transient response is another key point that requires a lot of in-depth studies. Therefore, future work will focus on the improvement of transient capability of the signal tracking strategy in the SSV.

## Figures and Tables

**Table 1:** Coefficients for oven-controlled crystal oscillator (OCXO) and temperature compensated crystal oscillator (TCXO).

Oscillator Type	$h_{0}$ (s^2^/Hz)	$h_{-2}$ (1/Hz)
OCXO	1.0 × 10^−21^	1.0 × 10^−20^
TCXO	2.51 × 10^−26^	1.0 × 10^−22^

**Table 2:** The requirements of bearable dynamics for onboard GNSS receiver of the lunar probe.

Dynamic Type	Sustainable Range
Velocity	0~12 km/s
Acceleration	0~15 g
Jerk	0~4 g/s

**Table 3:** The mission control sequence (MCS) of the lunar probe during the simulation time.

Phase	Time Span	Visibility of GPS Satellite	Orbital Maneuvering Status
I	0~50 s	Not in view	No maneuver is made
II	51~300 s	In view	No maneuver is made
III	301~310 s	In view	Make amaneuver with a ramp LOS jerk (the peak is 40 m/s^3^)
IV	311~320 s	In view	Make amaneuverwith a constant LOS jerk of 40 m/s^3^

**Table 4:** Input IMU parameters [27] for local frequency prediction in SDR.

IMU Parameters		MEMS IMU	Civil IMU	Tactical IMU	Navigation IMU
gyro errors	constant bias, °/h	300	50	1	0.01
accelerometer errors	constant bias, mg	10	1	0.1	0.01

**Table 5:** The Doppler estimation errors of four OL tracking schemes with a drift time of 270 s.

OL Tracking Schemes	Aided by MEMS IMU	Aided by Civil IMU	Aided by Tactical IMU	Aided by Navigation IMU
Maximum frequencyError (Hz)	1564.6000	260.6740	5.2246	0.0535
